# Individual differences in human gaze behavior generalize from faces to objects

**DOI:** 10.1073/pnas.2322149121

**Published:** 2024-03-12

**Authors:** Maximilian Davide Broda, Benjamin de Haas

**Affiliations:** ^a^Experimental Psychology, Justus Liebig University Giessen, Giessen 35394, Germany; ^b^Center for Mind, Brain and Behavior, Universities of Marburg, Giessen, and Darmstadt, Marburg 35032, Germany

**Keywords:** gaze, domain-general, face, object, individual differences

## Abstract

Individuals differ in where they fixate on a face, with some looking closer to the eyes while others prefer the mouth region. These individual biases are highly robust, generalize from the lab to the outside world, and have been associated with social cognition and associated disorders. However, it is unclear, whether these biases are specific to faces or influenced by domain-general mechanisms of vision. Here, we juxtaposed these hypotheses by testing whether individual face fixation biases generalize to inanimate objects. We analyzed >1.8 million fixations toward faces and objects in complex natural scenes from 405 participants tested in multiple labs. Consistent interindividual differences in fixation positions were highly inter-correlated across faces and objects in all samples. Observers who fixated closer to the eye region also fixated higher on inanimate objects and vice versa. Furthermore, the inter-individual spread of fixation positions scaled with target size in precisely the same, non-linear manner for faces and objects. These findings contradict a purely domain-specific account of individual face gaze. Instead, they suggest significant domain-general contributions to the individual way we look at faces, a finding with potential relevance for basic vision, face perception, social cognition, and associated clinical conditions.

## Significance

Individuals vary in their preference for where they look in a face, with some fixating near the eyes and others closer to the mouth region. These biases generalize from the lab to the world and are frequently discussed as clinically significant for social cognition. However, the mechanisms underlying individual face gaze are unclear. Analyzing over a million fixations to faces and objects from hundreds of observers, we show that individual fixation biases toward faces are not face-specific, but generalize to inanimate objects. People fixating closer to the eyes (or mouth) also fixate higher up (or lower down) on objects. We conclude that individual face fixation biases are at least partly driven by domain-general mechanisms of active vision.

Human individuals differ in the way they look at faces, including their tendency to fixate the eye region or lower down (1–3). These individual differences in face fixations are highly consistent (1), generalize from image viewing in the lab to real-life interactions (4), have a strong genetic component (5), and were proposed as clinical markers. Atypical face fixations have been reported for autism spectrum disorders (6, 7) and prosopagnosia (8–10). Specifically, it has been argued that these conditions are characterized by an avoidance of the eye region (6). However, contradicting evidence exists (11) and a recent study has shown that neurotypical variation in social anxiety and autism-like symptoms does not co-vary with eye-looking behavior (12).

Generally, gaze behavior is shaped by the constraints of the visual system. Visual acuity drops off from the fovea to the periphery (13, 14) and humans move their eyes to sample information from informative image regions at high resolution. This also shapes the way we look at faces. The optimal landing position of face-directed saccades is just below the eyes, according to a Foveated Ideal Observer model (15). While individuals with superior face recognition skills fixate close to this theoretical optimum (16), not everybody does. As mentioned above, the eye movements of some are biased toward the eye region while those of others consistently target the mouth or nose region. Forced fixation experiments investigated the effect of presenting faces at different heights relative to a fixated point. Presenting the face for a duration that is shorter than typical saccadic latencies allowed experimental control of the vertical retinotopic positioning of the face. Such experiments revealed that observers do best at recognition tasks when the forced fixation location corresponds to the location observers target spontaneously, regardless of whether this location adheres to the Foveated Ideal Observer model or not (1). This surprising finding may reflect individually shifted face templates in retinotopic coordinates (1, 4) which in turn may be linked to social cognition (17). That is, the reasons for individual differences in face gaze may be domain-specific.

An alternative hypothesis is that individual differences in face looking are due to domain-general idiosyncrasies, e.g., in the geometry of the visual field (1). The Foveated Ideal Observer model (15) predicts fixations just below the eyes based on the assumption of perfectly symmetric foveation. However, there are well-known canonical visual field biases, like the horizontal bias (perceptual and cortical resolution is better along the horizontal compared to the vertical meridian) and the vertical meridian asymmetry (perceptual and cortical resolution is higher along the lower compared to the upper vertical meridian) (13, 18). Crucially, previous research has also documented significant idiosyncratic visual field biases, with considerable perceptual consequences. Observers show idiosyncratic anisotropies in size perception (19), contrast sensitivity (20), and crowding (21). Such low-level, domain-general and idiosyncratic biases may shape individual eye movements in the same way foveation does in general (22).

Here, we set out to juxtapose the domain-general and face-specific hypotheses of individual differences in face-looking by testing a simple prediction. If individual differences in the vertical position of face fixations are influenced by domain-general visual field biases, they should generalize to fixations landing on inanimate objects. Alternatively, if individual differences in face fixations are determined by shifted face templates and/or social cognition alone, vertical fixation positions on faces should be independent of those on inanimate objects. We analyzed >1.8 million fixations towards faces and objects from over 400 observers freely viewing a set of static natural scenes (3, 23). Our findings replicate robust individual differences in preferred fixation locations for faces and, crucially, find that these differences generalize to inanimate objects. Interindividual differences in face fixations scale with the vertical size of a presented face in precisely the same way as expected for any other visual object. This contradicts a purely domain-specific account of individual face-looking.

## Results

### Consistent Fixation Biases within Faces and Objects.

Participants freely viewed 700 images of everyday scenes while we tracked their gaze. We collected an original dataset at Justus Liebig University Giessen, Germany (Gi2023, N = 252) and reanalyzed two pre-existing datasets to probe the robustness of our findings [Gi2019, N = 51, (24); Tr2020, N = 103, (25)].

**Fig. 1.**
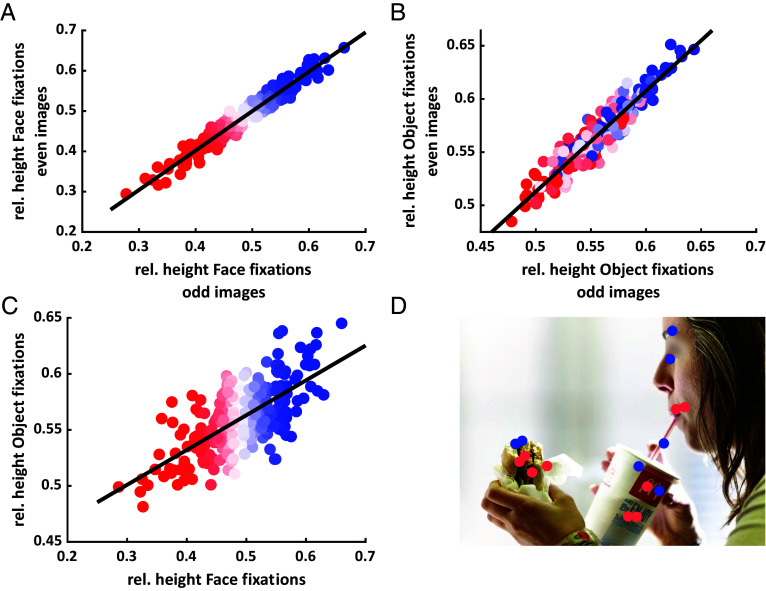
Vertical fixation positions in faces and objects. (*A* and *B*) Scatter plots for consistency correlations for vertical (*A*) face (*r* = 0.97, *P* < 0.001) and (*B*) object (*r* = 0.94, *P* < 0.001) fixation positions. (*A* and *B*) Each scatter point represents the average vertical fixation position of a single observer. Vertical fixation position was computed relative to the extent of the target along the vertical image axis, which coincided with the vertical meridian of the observer (chin and forehead rest; top-most pixel of the object corresponding to 1 and bottom-most to 0). For faces, only the inner face region was considered, and 0 and 1 corresponded to the bottom of the chin and hairline, respectively. (*C*) Correlation between the relative vertical positions of fixations landing in faces and objects (*r* = 0.69, *P* < 0.001). Each scatter point shows the average vertical fixation position of one observer within faces and objects. (*A*–*C*) The black lines depict the linear least-squares fit for each scatter plot. The color of scatter points always corresponds to the individual height of fixations in faces (red-blue from bottom to top). Note that the axis scales correspond to the relative height of fixations. In absolute terms, the spread on faces and objects was highly similar when considering target size (Fig. 3). (*D*) Examples of fixations landing on faces and objects for two observers (blue and red, respectively). Fixations in blue and red originate from participants who tended to fixate higher up and lower down, respectively. Blurring of the eye region for publication purposes only.

First, we extracted all fixations that landed on human faces or objects and determined their position relative to the vertical extent of the respective face or object. Then, we pooled all fixations of a given observer across all images and calculated the individual average of these vertical positions, separately for faces and objects. Our investigation presupposes that every individual has an average vertical fixation tendency which we can robustly estimate. We tested this assumption using split-half correlations across odd and even images, which confirmed highly consistent individual differences for vertical fixation positions on faces (*r* = 0.97, *P* < 0.001; Fig. 1*A*). Note that this does not preclude intra-individual variability around this individual average on a face-by-face or object-by-object level. Rather, a high split-half reliability indicates that our estimates of the individual average tendency were robust. The inter-individual spread of average face fixation locations reached from mouth to eyes (*SI Appendix*, Fig. S1), matching previous findings for isolated (1), static and dynamic scene-embedded (2, 3), and real-world faces (4). Importantly, we also found individual differences in vertical fixation positions on objects, which proved similarly robust, as indicated by a high split-half reliability (*r* = 0.94, *P* < 0.001; Fig. 1*B*). The inter-individual spread of average object fixation locations reached from about 50 to 70% of total object height (that is, the spread was confined to the second quarter from the top). Just as expected for domain-general visual field biases, we found equally consistent individual fixation biases along the horizontal, with high split-half reliabilities of horizontal biases for both, faces (*r* = 0.95, *P* < 0.001) and objects (*r* = 0.94, *P* < 0.001; see *SI Appendix*, Fig. S2). All of these biases were replicated in both pre-existing datasets (**SI Appendix*, Supplemental Results*). Additionally, both pre-existing datasets revealed moderate to strong test–retest reliabilities of individually preferred fixation locations (**SI Appendix*, Supplemental Results*).

### Fixation Biases Generalize between Faces and Objects.

Crucially, individual fixation preferences are generalized between faces and objects. Individual vertical fixation positions on faces and objects were highly correlated with each other (*r* = 0.69, *P* < 0.001; Fig. 1*C* and *SI Appendix*, Fig. S1), contradicting the hypothesis of a purely domain-specific mechanism. The same held for horizontal fixation positions, which were also highly correlated (*r* = 0.90, *P* < 0.001; *SI Appendix*, Fig. S2). These correlations between face and object-directed saccadic biases proved highly robust across a range of control analyses. Specifically, the correlation between individual fixation biases for faces and objects held for both replication datasets; for fixation heights on isolated larger faces; when controlling for estimated individual calibration errors; when controlling for potential motor biases, as indicated by individual average errors when saccading toward simple dot stimuli and when excluding highly overlapping objects (**SI Appendix*, Supplemental Results*).

### Scaling of Fixation Biases with Target Size.

If individual fixation heights are driven by visual field biases, their inter-individual spread may scale with the target size (26). Calibration biases, on the other hand, should be constant. Further, the relationship between the scale of vertical fixation biases and target size should generalize across objects and faces if the same, domain-general mechanism is at play.

**Fig. 2.**
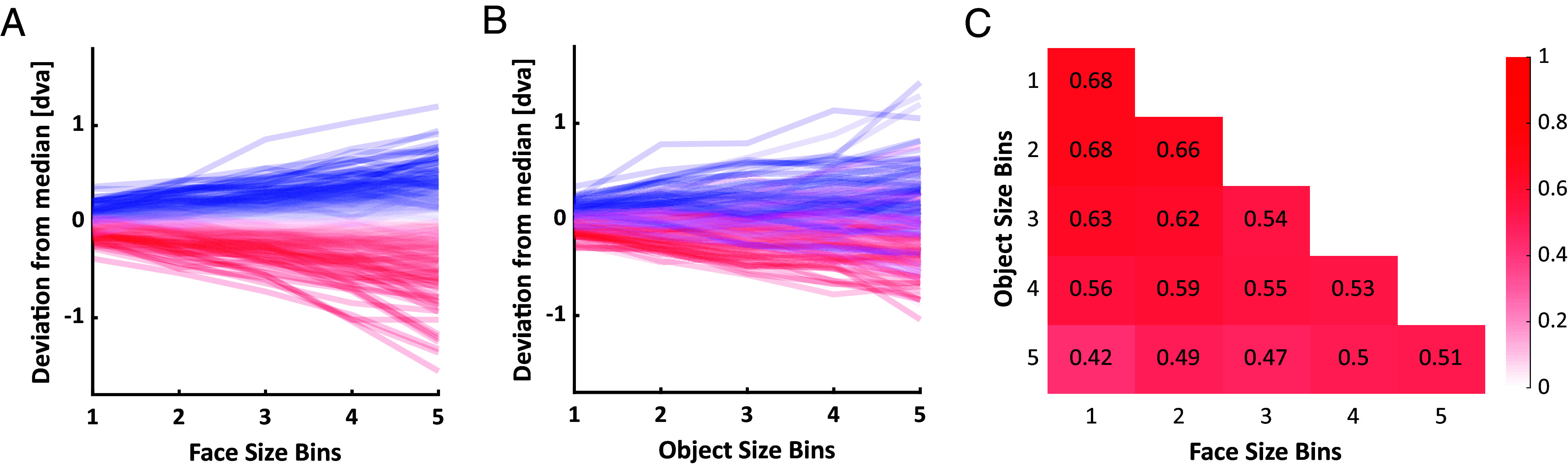
Scaling of individual differences with target size. (*A* and *B*) Each line shows the individual vertical deviation from the group median in degrees visual angle for one observer and across different (*A*) face and (*B*) object size bins. Faces and objects were divided into five bins containing an equal number of targets, based on their vertical extent. The individual deviation of vertical fixation positions from the group median was calculated separately for each target and then averaged for each observer and bin. (*C*) The heatmap depicts all intercorrelations between face and object size bins (all *r* > 0.4, all *P* < 0.001). Colors correspond to correlation values as indicated by the color bar to the right.

To test these hypotheses, we first quantified the spread of individually preferred vertical fixation positions around the group median for quintiles of target sizes. The inter-individual spread dramatically widened for both, larger faces, and objects (as confirmed by Levene’s test for differences in variance across quintiles; *P* < 0.001 for both, faces and objects). At the same time, relative inter-individual differences were preserved (as indicated by moderate to high correlations of individual differences across size quintiles; all *r* > 0.4, *P* < 0.001). As shown in Fig. 2, individual biases scaled with target size, but their direction and rank order were largely preserved.

**Fig. 3.**
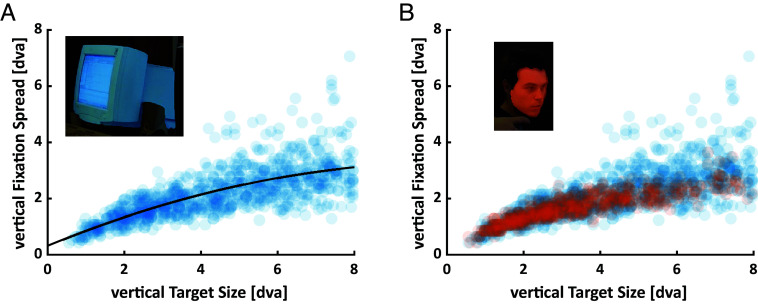
Scaling of inter-individual spread with target size. Each scatter point shows the estimated spread of individual vertical fixation positions for a given (*A*) object or (*B*) face as a function of its vertical extent. This analysis was limited to targets with a vertical extent <8 degrees visual angle and fixated by at least 30% of participants. Interindividual spread was estimated as five times the median absolute deviation of vertical fixation position, roughly corresponding to the 95% interval. (*A*) The black line shows the best-fitting second-degree polynomial to the size-spread relationship for objects (*R^2^* = 52%). (*B*) The size-spread relationship of faces (orange) was remarkably similar to that of objects (blue) and well described by the same, object-fitted polynomial (*R^2^* = 58%).

Next, we aimed to quantify the relationship between the scale of individual fixation biases and target sizes in absolute terms. We therefore estimated the spread of individual vertical fixation positions on a target-by-target basis. This analysis revealed a nonlinear relationship between vertical fixation spread and the vertical extent of objects, which was best fitted by a second-degree polynomial function (*R ^2^* = 52%) and showed some degree of heteroskedasticity. That is, the variability of the vertical fixation spread was higher among larger compared to smaller objects (Fig. 3*A*). Remarkably, the vertical spread of face fixations scaled in precisely the same way with the vertical distance between chin and hairline (Fig. 3*B*). Applying the polynomial which was fitted to object data to the spread of face fixations surpassed the noise ceiling (*R^ 2^* = 58%), presumably due to the lower degree of heteroskedasticity observed for faces. That is, the spread of vertical fixation positions followed the same non-linear function of target size, regardless of whether the target was an object or face.

### Intraindividual Consistency of Fixation Biases Generalizes between Faces and Objects.

We also tested whether individuals differ in the degree to which their fixation positions vary intra-individually. Again, a domain-general mechanism would predict that the level of intraindividual consistency generalizes between faces and objects. To test this hypothesis, we quantified the individual spread of relative fixation positions along the vertical and horizontal. Testing split-half reliabilities, we found that individual differences in this intraindividual spread were moderately consistent across odd and even images for both, faces (vertical: *r* = 0.70, horizontal: *r* = 0.67, both *P* < 0.001) and objects (vertical: *r* = 0.75, horizontal: *r* = 0.73, both *P* < 0.001). Just as expected for a domain-general effect, the amount of intraindividual spread was also moderately correlated between faces and objects (vertical: *r* = 0.53, *P* < 0.001, horizontal: *r* = 0.55, *P* < 0.001) as well as between the horizontal and vertical (faces: *r* = 0.28, objects: *r* = 0.66, both *P* < 0.001).

The amount of intraindividual spread appeared independent of the individually preferred vertical fixation position in faces. That is, the amount of intraindividual variance did not correlate with the tendency to look higher in the face (*r* = −0.07, *P* = 0.262) and visual inspection revealed no non-linear trends either. However, there was a weak but statistically significant negative correlation between higher average fixation positions in objects and intra-individual spread around that individual average (*r* = −0.23, *P* < 0.001). This may be driven by ceiling effects. Virtually all participants on average fixated the upper half of objects. Therefore, individuals at the upper (but not lower) end of the distribution had an average landing position close to the object edge, which cannot go along with a large intraindividual spread.

### Consistent Fixation Biases across Target Aspect Ratios.

The aspect ratio of fixated faces was significantly more homogeneous (SD = 0.30) than that of fixated objects (SD = 0.83), as confirmed by Levene’s test, *P* < 0.001. Therefore, we tested whether the correlation between vertical fixation biases on objects and faces was driven by objects with a similar aspect ratio to faces. We re-calculated the correlation between fixation biases toward faces and objects, separately for an aspect ratio highly similar or dissimilar to that of faces (i.e., within ±0.5 SD or beyond ±>2 SD from the mean aspect ratio of faces). Vertical fixation positions on faces were highly correlated with those on objects, regardless of their aspect ratios (high face similarity: *r* = 0.67, *P* < 0.001; low face similarity: *r* = 0.65, *P* < 0.001).

## Discussion

Previous research found consistent individual differences in preferred vertical fixation locations on faces that generalize from static (1, 3) to dynamic (2) scene viewing and real-world behavior (4). Here, we find that individual fixation biases are highly correlated across faces and objects, with about 50% of the explainable variance shared. This correlation held independent of target size and aspect ratio, was robust to controlling for calibration and motor biases and also held for the horizontal. Similarly, the intraindividual consistency of fixation locations generalized between faces and objects. Finally, the inter-individual spread of vertical fixation positions scaled with target size in precisely the same, non-linear manner for faces and objects. All of these findings are in line with the hypothesis of a shared, domain-general mechanism mediating individual fixation preferences on faces and objects and contradict a purely face-specific account.

The finding of consistent fixation biases on objects is remarkable given the variability of object shapes and the unknown geometry of their informative features. Presumably, the size of the present dataset (containing >400 observers and fixations on more than 3,000 annotated objects) helped to pick up the signal of individual fixation biases in the face of this diversity. Interestingly, individual averages of vertical fixation positions fell almost exclusively in the upper half of objects, which matches general visual field asymmetries along the vertical meridian, as objects will be shifted toward the more sensitive lower visual field (13, 18, 20). No comparable asymmetry was found for the distribution of individual face and object fixation averages along the horizontal (*SI Appendix*, Fig. S2). Also, the upward bias of object-directed fixations did not generalize to faces. Observers’ average fixation positions spread across the central face region, from mouth to eyes. Importantly, this difference between faces and objects was limited to the vertical intercept of the fixation distribution. The absolute spread of fixation positions scaled with absolute target size in precisely the same way for faces and objects. The observed intercept difference may be linked to the specific geometry of informative features in faces and may vary across classes of objects. Future research could try to identify further object classes with a stereotypical geometry of informative features that are frequently fixated.

Taken together, our results strongly suggest that the individual way we look at faces is at least in part shaped by domain-general mechanisms. This is at odds with purely domain-specific accounts, explaining individual face-gaze as a function of social cognition or retinotopic face templates alone (1, 17, 27). We do not yet know which domain-general mechanisms mediate individual fixation biases toward objects and faces. Previous studies have found both, the canonical visual field anisotropies described above (13), as well as idiosyncratic anisotropies in the functional anatomy of the retina (28), early visual cortex (19), and basic aspects of visual perception (19, 27). Specifically, human photoreceptor topography is highly variable (14) and may contribute to individually different zones of integration (28) as well as gaze behavior (26). Retinotopic maps in early visual cortex show quadrant-specific observer-idiosyncratic variation, which is associated with corresponding biases in size perception (19). Also, basic as well as high-level perceptual features, such as spatial frequency sensitivity and the perception of gender and age from faces can be spatially biased in idiosyncratic ways (27). Note that idiosyncratic low-level biases have been documented both, along the vertical and horizontal meridian (e.g., ref. 19), which matches our finding of individual biases in preferred fixation positions along both, the vertical and horizontal.

Our results suggest at least two independent mechanisms at play for individual fixation biases. Individual average fixation positions and the individual spread of fixations both generalized between faces and objects but proved mostly independent of each other. We speculate that spatial biases may be related to idiosyncratic asymmetries across polar angles (19–21), while the intraindividual spread of fixation locations may be linked to the individual slope of foveation along eccentricity (14, 21).

Future studies should decipher which domain-general biases shape the individual way we look at faces and how they interact with possible domain-specific factors, such as social cognition (17), experience with faces (16, 29), and visual field biases of neural populations with categorical preferences (30). Retinotopic face templates may be mediated by feature-location contingencies in neural tuning. Previous research investigating face recognition performance and neural tuning has found a preference for eyes in the upper parafovea and for mouths in the lower (31–33). This general tendency may vary somewhat across observers and contribute to individual vertical fixation positions (30). Throughout development, domain-general and face-specific factors may interact with gaze behavior in a self-reinforcing way. This would predict that with age, intraindividual variance in preferred landing positions decreases, while interindividual variance increases, a notion that can be tested in developmental experiments.

## Materials and Methods

### Datasets.

The present study analyzed three different free-viewing datasets: The Gi2023 dataset included fixation data from 251 participants. The Tr2020 dataset (25) involved 103 participants in two sessions, and the Gi2019 dataset (24) had 51 participants, also in two sessions. All studies used an EyeLink 1000 or EyeLink 1000 Plus eye tracker (see *SI Appendix* for more details).

The Justus Liebig Universität Fb06 Local Ethics Committee (lokale Ethik-Kommission des Fachbereichs 06 der Justus Liebig Universität Giessen) approved the study conducted in accordance with the declaration of Helsinki. All participants provided written informed consent before the experiment.

### Stimuli and Data Acquisition.

The stimulus set consisted of 700 natural scenes (https://www-users.cse.umn.edu/~qzhao/predicting.html) with labels and pixel masks for a total of 5,551 objects (23). For our analysis, we marked 3,294 masks pertaining to inanimate objects (i.e., excluding human bodies as well as animals). To accurately determine the relative position of face fixations, we created 2,055 additional pixel masks for faces, eyes, and mouths, with face masks extending from chin to hairline (3). All participants freely viewed 700 images in blocks of 100, each block starting with a 9-point calibration procedure. The image order was consistent across participants. Each trial started with a central fixation disk. Participants were asked to fixate the disk and press a button to trigger the presentation of an image for 3 s. For the Gi2019 and Tr2020 datasets, participants repeated the procedure with all (Gi2019) or a subset of 200 images (Tr2020), respectively. Images and image order were consistent across participants.

### Labeling.

All fixations with an onset time earlier than 100 ms after image onset or shorter than 100 ms in duration were excluded from further analyses. Fixations that fell on a target (face or object) were labeled as object or face fixations. We then computed the position of each face and object fixation along the vertical/horizontal, relative to the group median on the respective target and relative to the vertical and horizontal extent of the target (defined by the *top/bottom* and *left/right*-most pixels).

### Statistical Analyses.

We first computed the average vertical and horizontal fixation locations on faces and objects for each observer. We then tested the consistency of these individual averages across odd and even images, as well as their consistency across faces and objects using Pearson correlations. The intraindividual spread was quantified as the individual median absolute deviation (MAD) of vertical/horizontal fixation positions in faces and objects. Again, we used Pearson correlations to test the consistency of individual MADs across odd and even images, as well as across the horizontal and vertical, and the generalization of individual MADs across faces and objects.

### Target Size.

To investigate the effect of target size, we first binned all targets fixated by at least 30% of participants into size quintiles (according to vertical extent), separately for faces and objects. Then, we computed the deviation of the individually preferred vertical landing position from the group median for each bin and compared the group spread across bins using Levene’s test. We further computed correlations of individual deviations across faces and objects and all size bins. Next, we estimated the spread of fixation distributions as a function of absolute target size. For each target fixated with a vertical extent <8 degrees visual angle (dva) and fixated by at least 30% of participants, we estimated the spread as five MADs of the individual vertical fixations, roughly corresponding to 95% intervals. We then plotted the estimated spread as a function of vertical target extents and fitted a second-degree polynomial function to the resulting size-spread object distribution. Finally, we tested the goodness of fit of this object-fitted function for the size-spread relationship of faces.

### Aspect Ratios.

To test the potential role of target aspect ratios, we correlated individual fixation heights on faces separately with those on objects that have a similar aspect ratio to faces and those on objects that have not. We first determined the mean and SD of the aspect ratios (vertical/horizontal size) of all faces and then selected objects with an aspect ratio within ±0.5 SD of the mean face aspect ratio and objects with an aspect ratio more than two SDs above or below that of faces.

### Calibration Check.

To control for calibration errors, we computed the vertical/horizontal offset of fixations from the nominal center when participants pressed the button to initiate a trial (i.e., during the presentation of the central fixation disk). For each observer, we then computed the median vertical/horizontal offset across all trials as an indicator of calibration error. Additionally, we correlated individual calibration errors with individual vertical/horizontal fixation positions in faces/objects and partially correlated individual vertical/horizontal fixation positions in faces and objects, while controlling for individual calibration errors.

### Overlapping Objects.

We used the object annotations by J. Xu et al. (23), according to which objects can be contained within other objects. The main analysis treated such instances as separate objects and could evaluate a given fixation as falling onto different heights of multiple objects simultaneously. To probe the robustness of the results against this analysis choice, we ran a control analysis excluding all objects that overlapped more than 80% with other objects.

### Supplemental Datasets.

Analyses of vertical/horizontal fixation positions, their consistency, and generalization across target types were repeated for the Gi2019 and Tr2020 datasets in the same way as performed for the Gi2023 dataset. Retest-Reliability was assessed in both replication datasets by correlating the individual median of relative vertical/horizontal fixation positions for faces/objects between sessions on day 1 (700 images) and day 2 (Tr2020: 200 images, Gi2019: 700 images). Additionally, we computed the Retest-Reliability of calibration biases in the Tr2020 dataset (calibration data was not available for the Gi2019 dataset) and compared Fisher-z transformed Retest-Reliabilities for calibration biases with Fisher-z transformed Retest-Reliabilities values of relative vertical fixation positions for faces/objects using a modified version of the Pearson-Filon statistic (ZPF) (34).

## Supplementary Material

Appendix 01 (PDF)

## Data Availability

Original data have been deposited in osf.io (https://osf.io/y4dje/). Previously published data were used for this work (24, https://osf.io/n5v7t/ and 25, https://osf.io/ekvj4/).
