# Supplementary material for: Individual differences in human gaze behavior generalize from faces to objects
Source: Proc Natl Acad Sci U S A. 2024 Mar 12;121(12):e2322149121. doi: 10.1073/pnas.2322149121 (PMC10963009; doi:10.1073/pnas.2322149121)
Supplement: Supplementary file 1 — Appendix 01 (PDF) [file pnas.2322149121.sapp.pdf]

PNAS

Supporting Information for
Individual differences in human gaze behavior generalize from faces to objects

Maximilian Davide Broda & Benjamin de Haas

Maximilian Davide Broda
Email: Maximilian.Broda@psychol.uni-giessen.de

This PDF file includes:

Supporting text
Figures S1 to S3
SI References

Supporting Information Text

Free viewing Experiment

Subjects & Setup. We collected an original dataset (Gi2023) and re-analyzed two datasets previously published by our lab (Gi2019, Tr2020).

The Gi2023 dataset consists of fixation data from 251 participants (Age: M = 25 years; SD = 5; 180 females, two non-binary) acquired at Justus Liebig University Giessen (comprising a total of 805687 fixations on faces and objects). All participants had normal or corrected-to-normal vision. The Justus Liebig Universität Fb06 Local Ethics Committee (lokale Ethik-Kommission des Fachbereichs 06 der Justus Liebig Universität Giessen) approved the study conducted in accordance with the declaration of Helsinki. All participants provided written informed consent before the experiment. Participants placed their heads in a chin and forehead rest and viewed the stimuli described below at 34.3 x 25.7 degrees visual angle. The experiment was controlled via Psychtoolbox version 3.0.16 (1) and Matlab R2019a (Mathworks, Natick, MA, USA) on a Windows 10 PC. Gaze data were acquired using an EyeLink 1000 eye tracker (tower mount, SR Research, Ottawa, Canada) at a frequency of 1 kHz.

The Tr2020 dataset consists of fixation data from 103 participants (Age: M = 25 years; SD = 6; 72 females) who participated in two sessions with an average of 16 days in between, acquired at the Leibniz Institute for Psychology Trier (comprising a total of 497621 fixations on faces and objects). During both sessions, participants placed their heads in a chin and forehead rest and viewed the stimuli described below, while an EyeLink 1000 Plus eye tracker (desktop mount, SR Research, Ottawa, Canada) recorded their gaze. For details, see (2).

The Gi2019 dataset consists of fixation data from 51 participants (Age: M = 24 years; SD = 4; 40 females) acquired at Justus Liebig University Giessen (comprising a total of 351459 fixations on faces and objects). 48 participants completed a second session. During both sessions, participants placed their heads in a chin and forehead rest and viewed the stimuli described below, while an EyeLink 1000 eye tracker (tower mount, SR Research, Ottawa, Canada) recorded their gaze. For details, see (3).

Supplemental Results

Calibration Check. To estimate potential calibration errors, we analyzed the individual vertical/horizontal offset from a central fixation target presented between trials. This indicator of calibration bias was correlated with vertical fixation positions in faces (r = .49, p < .001) and objects (r = .51, p < .001) as well as with horizontal fixation positions (Faces: r = .81, p < .001; Objects: r = .73, p < .001). Crucially, partial correlations of fixation positions in faces and objects, while controlling for the estimated calibration bias, revealed marginally attenuated, but still strong correlations between vertical (r = .58, p < .001) and horizontal (r = .76, p < .001) fixation positions in faces and objects, showing that this relationship held independently of calibration errors.

Overlapping Objects. Excluding objects contained within or overlapping with other objects did not influence the correlation between vertical face and object fixation positions (r = .67, p < .001).

Supplemental Datasets. For the Tr2020 dataset, split-half correlations across odd and even trials replicated highly consistent individual differences for the relative vertical position of face (r = .97, p < .001) and object (r = .95, p < .001) fixations. Individual vertical fixation positions for objects and faces were highly correlated with each other (r = .69, p < .001). Furthermore, the re-test reliability of individual vertical fixation positions was high for faces (r = .82, p < .001) and objects (r = .72, p < .001). Similarly, split-half correlations across odd and even trials replicated highly consistent differences for the relative horizontal face (r = .94, p < .001) and object (r = .95, p < .001) fixation positions. Individual horizontal fixation positions for objects and faces were highly correlated with each other (r = .90, p < .001) and had moderate re-test reliability for faces (r = .58, p < .001) and objects (r = .50, p < .001). The re-test reliabilities of vertical (r = .40, p < .001) and horizontal (r = .35, p < .001) calibration biases were significantly lower than that of individual vertical/horizontal fixation biases in faces (vertical: r = .82; ZPF = -6.08, p < .001; horizontal: r =

.58; ZPF = -4.14, $p < .001$) and objects (vertical: $r = .72$; ZPF = -2.81, $p < .001$; horizontal: $r = .50$; ZPF = -1.63, $p < .001$).

For the Gi2019 dataset, split-half correlations across odd and even trials replicated highly consistent individual differences for the relative vertical positions of face ($r = .97$, $p < .001$) and object ($r = .92$, $p < .001$) fixations. Individual vertical fixation positions for objects and faces were highly correlated with each other ($r = .59$, $p < .001$). Furthermore, the re-test reliability of individual vertical fixation positions was moderate to high for faces ($r = .80$, $p < .001$) and objects ($r = .67$, $p < .001$). Similarly, split-half correlations across odd and even trials replicated highly consistent individual differences for the relative horizontal position of face ($r = .90$, $p < .001$) and object ($r = .93$, $p < .001$) fixations. Individual horizontal fixation positions for objects and faces were highly correlated with each other ($r = .87$, $p < .001$). Furthermore, the re-test reliability of individual horizontal fixation positions was moderate for faces ($r = .63$, $p < .001$) and objects ($r = .62$, $p < .001$).

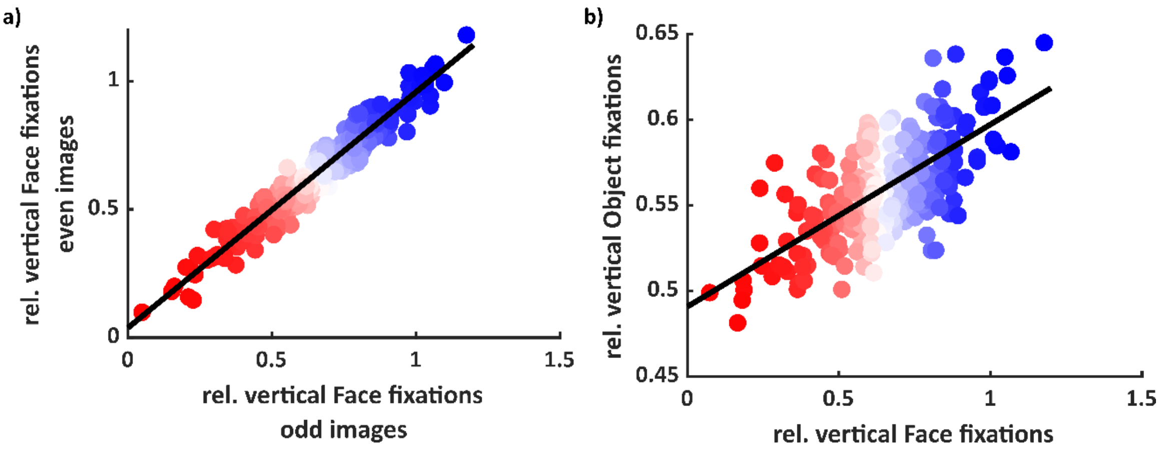

Figure S1. Vertical fixation positions relative to eyes and mouths.

(a) Scatter plots for consistency correlations for the vertical face ($r = .97$, $p < .001$) fixation positions scaled relative to the mean pixel position of mouths (0) and eyes (1). Each scatter point shows the average vertical fixation position of one observer within faces. Each scatter point shows the average vertical fixation position of one observer. (b) Correlation between the relative vertical position of fixations landing in faces relative to the eye and mouth region and objects ($r = .69$, $p < .001$). (a-b) The black lines depict the linear least-squares fit for each scatter plot. Blue and red scatter points show data from participants who tended to fixate higher up and lower down in faces, respectively.

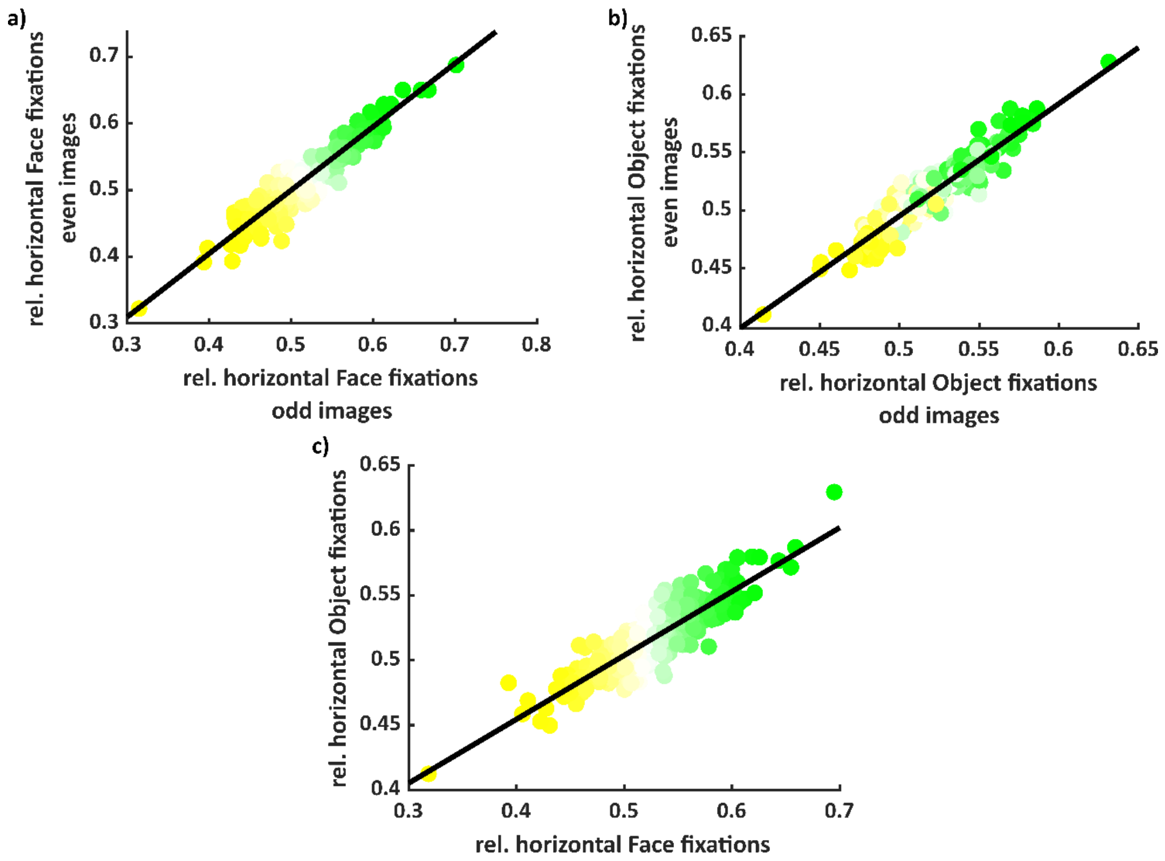

Figure S2 Horizontal fixation positions in faces and objects.

(a-b) Scatter plots for consistency correlations for horizontal (a) face (r = .95, p < .001) and (b) object (r = .94, p < .001) fixation positions. (a-b) Each dot represents the average horizontal fixation position of a single observer. The horizontal position was computed relative to the extent of the target along the horizontal image axis, which coincided with the horizontal meridian of the observer (chin and forehead rest; left-most pixel corresponding to 0 and right-most to 1). (c) Correlation between the relative horizontal position of fixations landing in faces and objects (r = .90, p < .001). Each scatter point shows the average horizontal fixation position of one observer within faces and objects. (a-c) The black lines depict the linear least-squares fit for each scatter plot. Yellow and green scatter points show data from participants who tended to fixate more to the left or more to the right in faces, respectively.

Saccade Recognition Task

Subjects & Setup. A subset of the Gi2023 sample (58 participants: Age: M = 25 years; SD = 6; 44 females) was invited back into the lab for a Saccade Recognition Task that roughly followed (4). Participants placed their head in a chin and forehead rest and viewed the stimuli described below at 16.23 x 16.23 degrees visual angle. The experiment was controlled via Psychtoolbox version 3.0.16 S1 and Matlab R2019a (Mathworks, Natick, MA, USA) on a Windows 10 PC. Gaze data were acquired using a tower mount EyeLink 1000 eye tracker (SR Research, Ottawa, Canada) at a frequency of 1 kHz. This study was approved by the local ethics committee at Justus Liebig University Giessen, and conducted in accordance with the declaration of Helsinki. All participants gave informed consent before the experiment. The Saccade Recognition Task was conducted three months after the free-viewing experiment on average.

Stimuli & Data Acquisition. Stimuli consisted of ten grey scale images of male faces that were selected from the SiblingsDB set (5). All faces were resized to an eye-mouth distance of 6 degrees visual angle, cropped to the inner face region and embedded in a black square with a

size of 16.23 degrees visual angle (Figure S3 b). Participants completed 180 trials that were split into six blocks, with re-calibrations in between. Each trial started with the presentation of a dot at one of 18 different locations with a mean distance of 15.23 degrees visual angle to the center of the screen (Figure S3 b). Participants had to saccade towards the dot and fixate within a radius of 1.5 dva of the dot to start the trial via key press. After a random delay of 500-1500 ms the stimulus was presented for 350 ms and then replaced by a 250 ms white noise mask. Participants were instructed to saccade towards the face as soon as it appeared. After the noise mask, a (constant) set of ten candidates was shown and participants could use the keyboard arrows and space bar to move a choice rectangle and confirm their choice, which was confirmed as correct or incorrect via a color change of the choice rectangle to green or red.

Estimating vertical landing positions and motor biases. We analyzed the first saccade of each trial during which fixation was not broken before image onset and computed the median vertical saccadic end positions across trials, separately for each individual. To test for a potential role of motor biases (vertical saccadic under-/over-shoot), we calculated the vertical error of saccades towards the target dots, which appeared at one of 18 possible locations in between stimulus presentations. Specifically, we analyzed the landing position of first saccades initiated after the display of the target dot, separately for each observer. Saccades were discarded if the landing position was outside a tolerance margin of 2 dva from the dot. For the remaining saccades, we computed the median vertical offset between landing positions and the target dot. 6 participants were excluded because they had fewer than 80% valid trials.

Statistical Analyses. Saccadic endpoint correlations were assessed by correlating the median relative vertical position of fixations within faces/objects in the free-viewing experiment with the median vertical position of saccadic endpoints in the recognition task. This analysis tested the generalization of individual differences in vertical fixation positions across stimulus types and sizes, tasks, and time. Furthermore, we tested a potential role of motor biases by correlating the estimated saccadic errors with the median relative vertical position of fixations within faces/objects in the free-viewing experiment.

Results. Vertical saccade landing positions in the recognition experiment correlated significantly with relative vertical fixation position for scene-embedded faces (r = .40, p = .003) and objects (r = .31, p = .027) during free-viewing (Figure *S3*), albeit lower than the consistency within the free-viewing experiment. Vertical offsets of the landing positions of saccades towards isolated dots (estimating a potential motor bias) were not correlated with the median relative vertical fixation positions within faces (r = .14, p = .317) or objects (r = -.06, p = .317) in the free viewing experiment.

a) b)

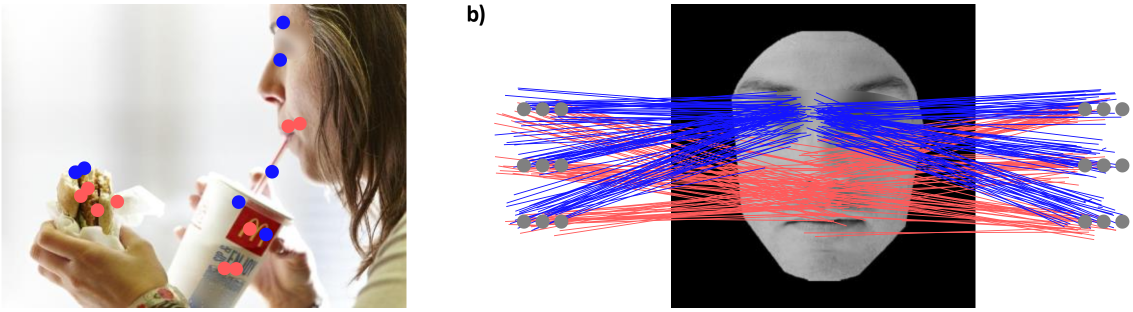

Figure S3. Vertical fixation positions in faces and objects.
(a) Examples of fixations landing on faces and objects for two observers (blue and red, respectively). Fixations in blue and red originate from participants who tended to fixate higher up and lower down, respectively. (b) Examples of saccadic start and landing positions from the Saccade Recognition Task. Participants started each trial at one of 18 locations outside the face

(indicated in grey). Each line shows the start and landing locations of a single saccade. Data in blue and red originate from the same two participants as those in (a). Blurring of the eye region for publication purposes only.

SI References

1. M. Kleiner, *et al.*, What's new in Psychtoolbox-3? *Perception* **36**, ECVP Abstract Supplement (2007).
2. M. Linka, B. de Haas, OSIEshort: A small stimulus set can reliably estimate individual differences in semantic salience. *J Vis* **20**, 1–9 (2020).
3. B. de Haas, A. L. Iakovidis, D. S. Schwarzkopf, K. R. Gegenfurtner, Individual differences in visual salience vary along semantic dimensions. *Proc Natl Acad Sci U S A* **116**, 11687–11692 (2019).
4. M. F. Peterson, M. P. Eckstein, Looking just below the eyes is optimal across face recognition tasks. *Proc Natl Acad Sci U S A* **109**, E3314–E3323 (2012).
5. T. F. Vieira, A. Bottino, A. Laurentini, M. De Simone, Detecting siblings in image pairs. *Visual Computer* **30**, 1333–1345 (2014).
